# Book Notices

**Published:** 1853-09

**Authors:** 


*A Valedictory Address to the Graduating Class of the Memphis Medical College*, delivered by H. V. Wooten, M.D., Professor of the Principles and Practice of Medicine, at the Commencement, Feb, 26, 1853.

*A Report on the Health and Mortality of Memphis* for the year 1852, by Charles Todd Quintard, M.D.

*Report of the Eastern Lunatic Asylum* in the City of Williamsburgh, Virginia, 1852–53.

*Essays on Asylums for Persons of Unsound Mind.* By John M. Galt, M.D., Superintendent and Physician of the Eastern Lunatic Asylum of Virginia, at Williamsburgh.

Dr. H. A. Ramsay's letter to Dr. James Bryan, on the Southern Negro, &c.

*An Address* delivered at the laying of the corner stone of the University of Nashville, April, 1853 By John A. McEwen, A.M.

*Galvanism*, its Application as a Remedial Agent. By C. H. Cleveland, M.D.

*Transactions* of the Thirteenth Annual Meeting of the Medical Society of Virginia, with the President's Address.

*A Review* of a Report of a Committee of the American Medical Association on the Permanent Cure of Reducible Hernia or Rupture. By George Heaton, M.D., Member of the Mass. Med. Society, &c.

Many of these works have been on our table for some time, and should have been noticed before.

Dr. Wooten's address consists of an examination of the elements of character necessary to success in the practice of medicine, or, indeed, in any profession in life. The style and train of thought is appropriate to the occasion. In the closing paragraph the Doctor says:

"No great end was ever attained without a great aim. Then set the mark of your destinies high, and march energetically, prudently and firmly up to it and you shall not fail. Indeed, you must listen to no such word as fail. Human life, when extended to its utmost limits, is but a span, and to be successful in the achievement of any great purpose, will admit of no waste or misapplication. But you are, perhaps, told, that it is useless now to enter upon a course of honorable ambition, because your profession is so heavily crowded with competition. It is, indeed, true, that in numbers, the field of labor is amply supplied. But when was it otherwise? And will you hesitate and falter, on that account? Suppose it were not so. If there were no competition, whence would triumph derive its glory? The honor of success is always measured by the magnitude and difficulty of the enterprise. Look at the hosts of good and useful, and great men, whom you may see, in almost every direction, who have wrought out their own high characters. Did they ever shun competition, or quail before its advances? Did you ever know one of them to wait for an opening? On the contrary, wherever he may spring up, the man who has the legitimate elements of success in his character, will be seen to approach nearer and nearer to the front of the column, and engage more and more heartily, in the strife for honorable advancement. Competition is the true school of efficiency and excellence, and the man who prefers to work without it, is like the school-boy who would study without a teacher. He who has not within himself, those elements of character, which will enable him to succeed, in the midst of competition, would not succeed under any circumstances, even of his own choosing. I speak of legitimate and permanent success.

But there is another matter to be remembered in this connection. Men are mere transitory beings. Those who, by years of assiduous labor, have acquired a reputation, are not to be your competitors; you are to compete with those of your own age, and who start even with you in the race. Older men are before you, but they must have their day, and pass away as the grass of the field. Be not impatient but wait and labor. Be steadily at the pool, and ready for the troubling of the waters. The men who seem now to crowd up so completely the avenues to success before you, will soon be ready to resign to you their places.—But while waiting for them, you must not, for a moment forget, that when obtained, these places to be held, must be *filled*, not merely *occupied*."

---

Dr. Quintard in his report on the health and mortality of Memphis, after alluding to the increased longevity of the present period, shows, that this increase is not due alone to the progress of civilization, but to a great extent, at least, to the progress of medicine. It is shown by the records of the Hotel Dieu, of Paris, that anterior to the great French revolution, the number of deaths to cases treated was one in three and a half, or nearly thirty per cent. In 1816 they were reduced to one in four and a half, and the diminution of mortality has been constantly progressive up to 1840, to which time the tabular returns are completed. The proportion of deaths has diminished two-thirds while the time of treatment in 1838 was just one half of what it was in 1816. The records of American Hospitals lead to the same conclusions. The mortuary statistics of Memphis are then examined carefully and thoroughly, and the conclusion arrived at, that the records of the board of health are valueless.

---

Dr. Galt, the superintendent of the Eastern Lunatic Asylum, of Virginia, and the author of the essays on asylums for the insane, had presented a previous series of papers on that subject; the present was prepared by the direction of the Association of Medical Superintendents of American Institutions for the Insane.

---

Dr. Ramsay's letter contains an exposition of his views on the peculiar physiological character of the negro, cautiously and rather timidly, as it seems to us, expressed. The following are his conclusions:

"1st. That the crossing of the same species, disconnected by blood relationship, in man, animals and fowls, is essential to their perpetuation.

2d. That the crossing of the same species, proximately connected by blood, has a deleterious influence upon them.

3d. That the crossing of the white and negro might merge the black race into the white, but it would be infinitely worse for the negro.

4th. That the negro is physically superior to the mulatto, while the latter has more intelligence and prettier features.

5th. That the amalgamation is objectionable, under any circumstances.

---

We were agreeably surprised and interested in Dr. Cleveland's pamphlet on galvanism and its application as a remedial agent. It is a compend of the opinions of many men, eminent in the medical profession, upon the value and method of using this mysterious but powerful agent.

We have always believed, that properly applied in accordance with scientific principles, it would be found to be most invaluable as a curative agent, but the apparatus ordinarily in use is defective. If any benefit is derived from it, it is purely accidental. The electro-magnetic, and magneto-electric machines, all of them, so far as we know, produce a series of shocks. Whatever be the quantity of electricity used, it should be of a low intensity; in the opinion of most writers, a single pair of plates being sufficient. This will produce no shock and no immediate sensible effect.

We quote the rules which our author gives for the application of galvanism :

"The writers already quoted have expressed themselves so distinctly, positively, and uniformly, in regard to the manner in which galvanism should be applied, that nothing remains to be done on that point but to *sum up* their statements in a concise form. But there is *one* very important matter,—in fact one that lies at the foundation of all correct and scientific use of this agent, that seems to have been frequently overlooked. I refer to the *kind* of galvanism employed, and especially whether of the *intense* or *destructive* kind, or otherwise.

As some, who are not practical chemists, do not appear to perceive wherein this distinction consists, I will briefly refer to the views of a few eminent chemists on this point.

*Silliman*, in the *First Principles of Chemistry*, presents the following proposition :—

'*Quantity* and *Intensity.*—We learn the remarkable fact, that no matter how much we may increase the number of members of the voltaic circle, the *quantity* of electricity passing in the current, is equal only to that evolved by a single cell. But the current which has passed through a number of similar cells, has acquired an *intensity* exactly proportioned to the number. Thus, *no single cell*, however large, would ever afford electricity of a *tension* sufficiently high to decompose water, or give the slightest shock to the animal frame.'

*Smee*, in his *Elements of Electro-Metallurgy*, p. 10, Am. edition, says :—'A hundred batteries may be conjoined, but no more electricity is obtained ; for the same *amount* of electricity passes as when one cell is used. Now, however, this same *amount* can pass through a much greater resistance, for it would seem as if, at every alternation of the battery, the electric fluid obtained a *push* to overcome any obstacle afforded to its passage.'

*Brande* says, (page 213, vol. 1, of his *Manual Chemistry*,) that the *intensity*, as shown by *Faraday*, in his researches, is in proportion to the *number* of plates, while the *quantity* is dependent upon the *extent* of the plate. The same position is taken by

*Sir H. Davy*, in the *Philosophical Transactions* for 1840. *Hoblyn* says, page 89 :—' It is necessary to point out the distinction between *quantity* and *intensity*.' *Gray* says, page 86, that —' Quantity refers to the *amount* of electric fluid set in motion ; *tension*, or *intensity*, to the energy with which the current is impelled.'

Although authors have been thus plain in pointing out the source of the *destructive* element in galvanism, yet, neither those who have invented, manufactured, or applied the instruments in use by the profession, seem to have kept this distinction clearly in view. Belts, chains, and other modifications of the simple galvanic battery, have been devised, and the first idea seems to have been, to make the surface of the single pair *quite small*, so that but little *amount* of the agent would be generated, and then to increase the *intensity* or *destructiveness* of this agent, by uniting as large a number of these pairs of metals as possible in the single circle. These are very powerful to destroy, to decompose, but cannot impart any great amount of healthful energy to the part to which they are applied.

In the selection of an apparatus, the first thing is to get one that has but *one element*—that is but one plate of the metal which is the most readily acted upon. This plate should be sufficiently large to generate the *amount* of galvanism required, and that amount may be diminished at pleasure, by cutting off a part of the current either from the whole surface of the plate, or from a part of its surface.

The apparatus should be so arranged, that the part to be acted upon can be kept under the *continued* influence of the agent, for the desired length of time. Thus we avoid the *reaction* and *exhaustion* which follow when a stimulant has been powerful, applied but a short time, and then suddenly withdrawn.

The apparatus should be simple in its construction, so as not to be liable to become deranged while not under the eye of the physician.

To increase the vital activity and energy of any organ, and to impart the power of *motion* in an organ that has become more or less paralysed, the apparatus should be so arranged, that the *positive* plate, or the copper, should be *above*, or toward the *origin* of the nerves which are distributed to that organ, and the *negative*, or zinc plate, should be on the opposite side, or at the *extremities* of the nerves.

This rule is based on the fact, that all *motion*, or *increased action* in any organ, originates in an impression received from the brain, which impression passes *from* the nervous centre *to* the extremities of the branches of the nerves.

To rouse *sensation* in any organ, as the sense of hearing, of sight, of touch, etc., the position of the plates should be reversed, so as to cause the galvanic influence to pass *from* the *extremities* of the nerves to their origin. This rule is based on the fact, that our nerves of *sensation* are acted apon by *external* impressions, and that these impressions are carried *from* the *extremities* of the nerves of sensation to the nervous centre.

Thus in each of the above cases, the galvanic influence is made to traverse in the direction of the *vis nervosa*. In applying the battery as last directed, great care should be used, as if two or more elements be used, or, in other words, if *intense* galvanism is applied, great pain will be felt, and irremediable harm may be done.

In making use of galvanism as a therapeutic agent, it should not be relied on to the exclusion of all other treatment; neither should a cure of the disease for which it is applied, be anticipated in a miraculously short space of time. Disease in any organ produces a change in the condition and structure of the organ diseased, and the time must be allowed for the processes of absorption and deposition necessary to bring the organ back to its normal condition. Galvanism, when properly applied, will be found of great value in hastening these processes; yet it will not do to apply it of such power as to *destroy* the organ from which we wish to remove the abnormal accumulations, or even to carry the action of that organ beyond the condition of *health*. As galvanism possesses great power over the animal economy, therefore it is imperatively necessary that patients to whom it is applied, should be immediately under the care and attention of a skillful physician. Any agent that is powerful, must be dangerous when applied in an improper manner; and one possessing the power to decompose water, and even the most intractable metals, should no more be placed beyond the control of those who fully understand its power and mode of action, than the locomotive should be allowed to start upon the track without being accompanied with a well instructed engineer."

The manner in which Dr. Cleveland uses this agent, is by placing plates of different kinds of metal connected by a steel spring, so that in completing the circuit, the current shall pass through the desired organ.

---

The transactions of the Medical Society of Virginia contain only the address of the president, Dr. Beale, and the minutes of the meeting. The society has made a beginning in the accumulation of a library, an herbarium, pathological museum, etc.

The following was unanimously adopted:

*Resolved*, That a committee of three be appointed, whose duty it shall be to draft a plan for the creation of a fund for the relief of decayed physicians, their widows and orphans, and that the committee shall report at the next annual meeting.

Medicine in Virginia, as elsewhere, it seems, is not remunerative. Men give to the profession the best of their lives, and when no longer able to engage in its active duties, they too often find that their labor has been for nought—while administering to the physical necessities of others, their own pecuniary necessities have been neglected. J.

